# Temperature Dependence of Light Hydrocarbons Sorption and Transport in Dense Membranes Based on Tetradecyl Substituted Silicone Rubber

**DOI:** 10.3390/membranes13020124

**Published:** 2023-01-18

**Authors:** Alexander O. Malakhov, Stepan E. Sokolov, Evgenia A. Grushevenko, Vladimir V. Volkov

**Affiliations:** A.V. Topchiev Institute of Petrochemical Synthesis, Russian Academy of Sciences, Leninsky Prospect 29, 119991 Moscow, Russia

**Keywords:** poly(tetradecyl methyl siloxane), light hydrocarbons, sorption, diffusion, permeation, selectivity, temperature dependence

## Abstract

Solubility-selective polymer membranes are promising materials for C_3+_ hydrocarbons removal from methane and other permanent gas streams. To this end, a dense solubility-selective membrane based on crosslinked poly(tetradecyl methyl siloxane) was synthesized. Sorption of methane, ethane, and *n*-butane in the polymer was measured in the temperature range of 5–35 °C. An abnormal temperature dependence of sorption was detected, contradicting the generally accepted view of sorption as an exothermic process. In particular, methane shows minimal sorption at 5 °C. The abnormal temperature behavior was found to be related to crystallization of the alkyl side chains at temperatures below ~10 °C. Gas permeability determined by sorption and permeation methods are in reasonable agreement with each other and decrease in the order *n*-C_4_H_10_ > C_2_H_6_ > CH_4_. The solubility of these alkanes changes in the same order indicating that poly(tetradecyl methyl siloxane) is indeed the sorption-selective membrane. The diffusivities and permeabilities of studied alkanes declined with decreasing temperature, whereas the *n*-C_4_H_10_/CH_4_ permselectivity increases with decreasing temperature, reaching a value of 23 at 5 °C.

## 1. Introduction

The separation of C_2+_ hydrocarbons from natural gas is usually realized by cryogenic distillation, which is an energy-intensive operation. Membranes are considered a lower-energy alternative, permitting the separation of natural gas into two streams, one enriched by light hydrocarbons (C_2+_) and the other by methane. Solubility-controlled (sorption-selective) rubbery and glassy polymer membranes are promising materials for C_2+_ removal [1,2,3,4,5,6]. Among amorphous rubbers, polydimethylsiloxane (PDMS) is a commercially available membrane for recovering condensable gases C_3+_ from their mixtures with permanent gases. It is the most permeable rubber polymer, but its disadvantages are poor mechanical properties and modest selectivity [7,8,9,10]. Therefore, current research is focused on improving the mechanical properties of PDMS, as well as on obtaining superior selectivity by physical or chemical modification of this and other rubbers [11,12,13].

The modification of PDMS by substituting one of the methyl groups for the *n*-alkyl side chain is one way to increase the selectivity of separation of C_3+_ hydrocarbons and permanent gases, such as methane. The introduction of *n*-alkyl chains of relatively short length (*n* ≤ 8) leads to a marked decrease in hydrocarbon permeability and a certain increase in selectivity [7,14,15,16]. Thus, Stern et al. [14] obtained that the methane and propane permeability through poly(octyl methyl siloxane) (POMS) is more than 4 times lower compared to PDMS, and the propane/methane selectivity of POMS slightly exceeds that of PDMS (6.5 vs. 5.9 at 35 °C). Schultz and Peinemann [7] obtained higher *n*-butane/methane selectivity of the POMS composite membrane compared to the PDMS composite membrane (12 vs. 5 at 30 °C) in mixed-gas conditions. Mushardt et al. [16] obtained an even higher *n*-butane/methane selectivity for the composite POMS membrane, ~22 at 20 °C when separating binary *n*-butane/methane mixtures. A more than twofold increase in the *n*-butane/methane selectivity of POMS over PDMS was obtained in [17] for multicomponent gas permeation (16.5 vs. 7.6 at 25 °C).

It is known that *n*-alkyl side chains in comb-like polymers are able to crystallize if they exceed a certain critical length *n_crit_*. This phenomenon was observed in various comb-like polymers, such as *N*-alkylated polyamides [18] with *n_crit_* = 13, poly(*n*-alkyl acrylates), poly(*n*-alkyl methacrylates) [19,20] with *n_crit_* = 9–10, and poly(*n*-alkyl itaconates) [21] with *n_crit_* = 12. At *n* > *n_crit_*, the melting point (*T*_m_) for a given polymer increases as the side-chain length increases. Paul and coworkers [19,20,22] found an anomalous gas permeation behavior of poly(*n*-alkyl acrylate)s and poly(*n*-alkyl methacrylate)s, namely, gas permeability increases by two orders of magnitude, and solubility rises about threefold with a change in the morphology of the side-chain from crystalline to amorphous upon melting. The same phenomenon, the crystallization of side chains, was found in poly(*n*-alkyl methyl siloxane)s for sufficiently long alkyl substituents (*n* ≥ 9) [23].

In our recent work, the crystallization of side-chains in crosslinked poly(*n*-alkyl methyl siloxane)s with *n* = 10, 12, and 14 was detected, and an increase of the *n*-butane/methane selectivity with alkyl length was revealed [24]. In particular, the selectivity for the separation of an eight-component light hydrocarbon mixture through a poly(*n*-decyl methyl siloxane) membrane at 25 °C was 18.8 compared with values of 16.1 for POMS and 7.5 for PDMS membranes. In addition, the temperature dependences of solubility and permeability of *n*-butane and methane in poly(*n*-decyl methyl siloxane) were studied. It was shown that the decrease in temperature from 50 to 0 °C gave rise to the *n*-butane/methane selectivity increase from 9.3 to 60 for individual gases, and from 8.3 to 34 for mixed gases [25].

This paper reports a study of hydrocarbon (CH_4_, C_2_H_6_, and *n*-C_4_H_10_) sorption, diffusion, and permeation through a poly(tetradecyl methyl siloxane) (PTDMS) membrane, focusing on the temperature dependence of gas solubility in the range 5–35 °C, which includes the melting point of the crystalline side chains.

## 2. Materials and Methods

### 2.1. Materials

Poly(methylhydrosiloxane) with *M_n_* = 1900 g/mol (ABCR, Karlsruhe, Germany), Karstedt’s catalyst (platinum complex of 1,3-divinyl-1,1,3,3-tetramethyldisiloxane in xylene, Sigma-Aldrich, St. Louis, MO, USA), 1-tetradecene (92%, Sigma-Aldrich, St. Louis, MO, USA), cross-linking agent 1,7-octadiene (98%, Sigma-Aldrich, Munich, Germany), and *n*-hexane (99%, Chimmed, Podolsk, Russia) were used for the synthesis of PTDMS. Helium (99.995%), methane (99.99%), ethane (99.95%), and *n*-butane (99.95%) were used for sorption and permeation measurements.

### 2.2. Membrane Preparation

The casting solution was prepared by mixing 3 wt% poly(methylhydrosiloxane) solution in *n*-hexane with a 15 wt% solution of 1-tetradecene in *n*-hexane. PTDMS, poly(siloxane) with tetradecyl side-chains, was obtained by a hydrosilylation reaction with 1-tetradecene in the presence of Karstedt’s catalyst at 60 °C for 2 h:

~[HSiCH_3_O] ~ + CH_2_=CH–(CH_2_)_11_–CH_3_ → ~ [C_14_H_29_SiCH_3_O]~

Afterward, a 10 wt% solution of the cross-linking agent (1,7-octadiene) in *n*-hexane (mole ratio of 1-tetradecene/1,7-octadiene = 95/5) was added into the reaction mixture (the time of the crosslinking 1 h, *T* = 60 °C). PTDMS membranes were obtained by pouring the casting solution into a stainless-steel net, which was fixed on a Teflon surface, with subsequent drying at 60 °C until a constant weight was reached. For sorption measurements, PTDMS films with an average thickness of 690 ± 20 μm were prepared, for permeability measurements, and films with a thickness 83 ± 2 μm were obtained.

### 2.3. Sorption Measurement

The magnetic suspension balance XEMIS (Hiden Isochema, Warrington, UK) was used to measure the sorption and desorption isotherms of CH_4_, C_2_H_6_, and *n*-C_4_H_10_ on the polymer sample. This system is able to perform sorption measurements while increasing the gas pressure from 0 to 10 MPa and for temperatures ranging from 273 to 373 K. The microbalance has a long-term stability of ±1 μg with a weighing resolution of 0.2 μg. The sample temperature was monitored throughout the experiment, and its variation was minimal (<0.3 °C). With the gravimetric method, the so-called reduced mass of sorbate Ω is measured at a given temperature and gas pressure [26]. The reduced mass differs from the adsorbed mass by a buoyancy correction:

(1) $$\Omega =m_{a}-\rho_{gas}V=m_{a}-\rho_{gas}\left(V_{sk}+V_{a}\right)$$

Here $\rho_{gas}=\rho_{gas}(p,T)$ is the gas density at pressure *p* and temperature *T*; *V* is the sample volume, which consists of two components: the true (skeletal) volume of the sorbent $V_{sk}$ and the adsorbed volume $V_{a}=m_{a}/\rho_{a}$. The skeletal volume of the sample was determined using a preliminary helium adsorption experiment. The density of the adsorbed substance $\rho_{a}$ is not available from gravimetric data. The standard assumption that the sorbate is in a liquid-like state was used. For *n*-butane, the sorbate density was assumed to be equal to the saturated-liquid density [27] at the temperature of the experiment; for methane and ethane, the density of a hypothetical liquid sorbate was calculated according to Saha’s recipe [28]:

(2) $$\rho_{a}=\rho_{b}\exp\left(-\alpha (T-T_{b})\right)=\rho_{b}\exp\left(T_{b}/T-1\right)$$

where *α* = 1/*T* is the isosteric coefficient of the expansion of the adsorbed volume, *ρ_b_* is the density of liquid adsorbate at the normal boiling point *T_b_*.

Expressing the mass of adsorbate from Equation (1), the following expression for absolute adsorption of gas is obtained:

(3) $$\;\frac{m_{a}}{m_{0}}=\frac{\Omega /m_{0}+\rho_{gas}/\rho_{sk}}{1-\rho_{gas}/\rho_{a}}$$

where *m*_0_ is the dry mass of the sorbent in a vacuum, $\rho_{sk}=m_{0}/V_{sk}$ is the skeletal density of the polymeric sorbent ($\rho_{sk}=0.942\pm 0.001\;$ g/cm^3^ at 25 °C). Gas sorption *C* in polymers is usually expressed in cm^3^(SPT) per 1 cm^3^ of polymer, that is,

(4) $$C=\frac{m_{a}}{m_{0}}\frac{\rho }{M_{a}}22414$$

where *ρ* is the bulk (geometrical) density of the polymeric film (g/cm^3^), *M_a_* is the molar mass of the adsorbate (g/mol). The bulk density at 25 °C was evaluated by the buoyancy method as

(5) $$\rho =\rho_{\mathrm{liq}}\frac{m^{\mathrm{air}}}{m^{\mathrm{air}}-m^{\mathrm{liq}}}$$

where *m*^air^ and *m*^liq^ are the masses of the polymer sample in air and in a nonwetting liquid, respectively. Water, which sorbs negligibly on siloxane polymers, was used as a nonwetting fluid. A density value of *ρ* = 0.889 ± 0.005 g/cm^3^ was obtained.

The polymer sample was evacuated at 40 °C for one day before starting measurements. The sorption isotherms for each gas were obtained by averaging the data over three cycles of sorption–desorption. The time of reaching the equilibrium value of the sorbate mass at a given gas pressure ranged from 1 to 3 h for the studied hydrocarbons.

It was found that the hydrocarbon transport in PTDMS follows Fickian diffusion (the sorbed mass is proportional to *t*^1/2^ for small values of time *t*). The diffusion coefficients of gases in the polymer film *D_s_* and *D_d_* were determined from the sorption-time and the desorption-time curves, respectively. Specifically, the penetrant diffusivities have been calculated as

(6) $$D_{s,d}=0.04919{L^{2}/t}_{0.5}$$

where *L* is the membrane thickness, $t_{0.5}$ is the sorption (desorption) half-time, that is, the time corresponding to $m_{a,t}/m_{a,\infty }=0.5$ ($m_{a,t}$ is the mass of penetrant sorbed at time *t*, $m_{a,\infty }$ is the asymptotic equilibrium value of $m_{a,t}$) [29]. The pressure dependences of the mean diffusion coefficients $D=\left(D_{s}+D_{d}\right)/2$ were fitted to a smooth spline function using Mathcad and extrapolated to zero pressure:$D_{0}=\underset{p\to 0}{\lim}D(p)$. The experimental accuracy in determining the diffusion coefficients was ±6%.

### 2.4. Permeability Measurement

The gas permeability of the membranes was measured by a constant-volume/variable-pressure method (HZG Gas and Vapour Permeability Test Unit, Geesthacht, Germany). The feed membrane was 150–1125 mbar, and the permeate pressure was maintained at less than 10^−4^ mbar. The steady-state permeability coefficient was calculated using the relation:

(7) $$P=\frac{VL}{At\Delta p}$$

where *V* is permeate chamber volume, *L* is the membrane thickness, *A* is the membrane area, and Δ*p* is trans-membrane pressure. The experimental error of the measurements of *P* is ±5%. The values of $P_{0}=\underset{p\to 0}{\lim}P$ were determined by extrapolating plots of ln *P* versus *p* to zero pressure at 5, 10, 15, 20, and 30 °C.

### 2.5. Thermal Analysis

Differential scanning calorimetric studies were carried out using a Mettler DSC823 instrument (Mettler Toledo GmbH, Greifensee, Switzerland) in argon atmosphere in temperature intervals from −100 °C to 50 °C with a heating rate of 10°/min. Thermal transition behavior was recorded during the first heating.

## 3. Results and Discussion

### 3.1. Sorption Isotherms

**Figure 1:**
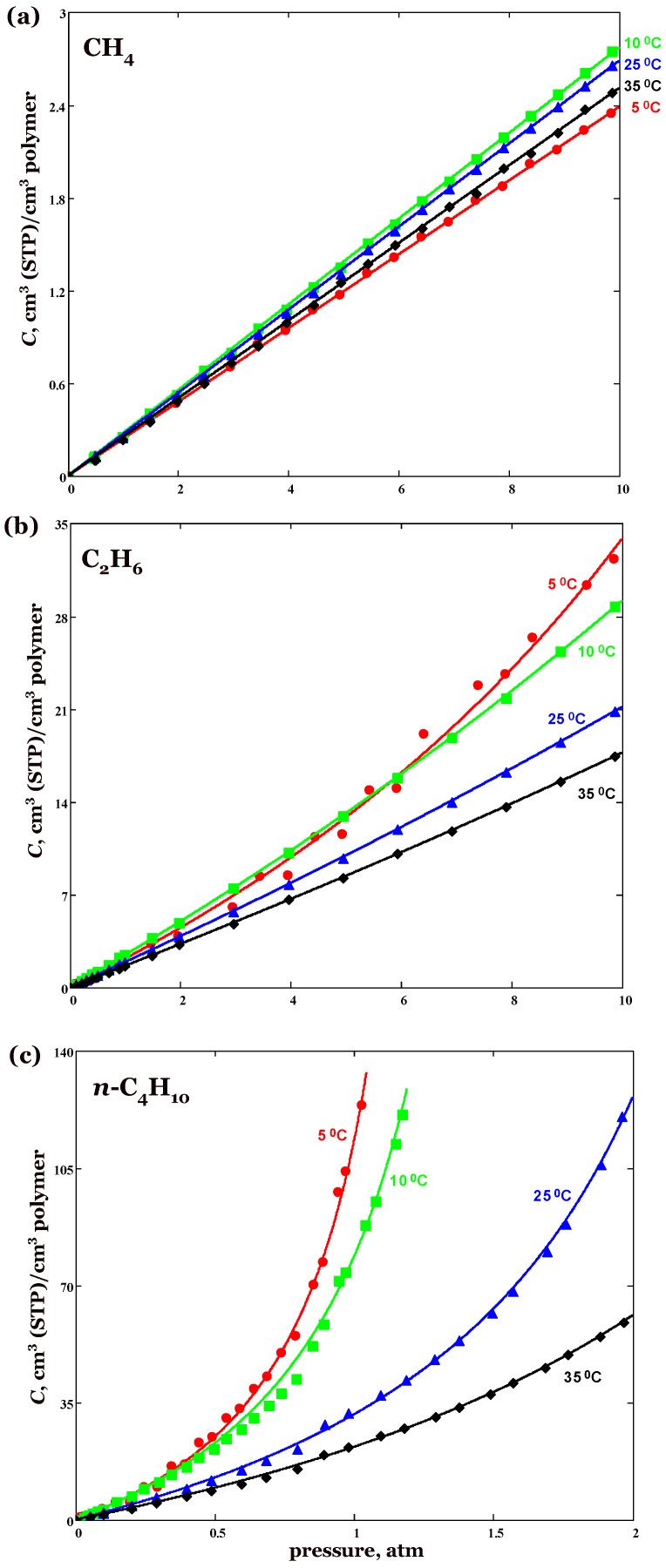
Methane (**a**), ethane (**b**), and butane (**c**) sorption isotherms in PTDMS silicone rubber at the temperature range 5–35 °C. Points—experimental data, lines—approximation of the experimental data by Equation (8); in the case of methane the linear function of gas pressure was used.

Hydrocarbon sorption isotherms in silicone rubber are shown in Figure 1. Methane sorption isotherms are linear over the range of pressure (up 10 atm). Ethane sorption isotherms are almost linear at ambient temperature, but become convex toward the pressure axis at lower temperatures. Butane sorption isotherms are convex toward the pressure axis over the entire temperature range (5–35 °C). Such isotherms (type III of the IUPAC classification) were observed for the sorption of C_3+_ in PDMS rubber [30]. Sorption isotherms, that is, the dependence of the equilibrium concentration *C* of the penetrant in the polymer on the pressure *p* of the penetrant in the gas phase, were approximated by the equation

(8) $$C(p)=\frac{S_{0}p}{1-bp}$$

where parameter *S*_0_ is the solubility coefficient *S* = *C*/*p* in the limit *p*→0, that is, the Henry’s law constant, which depends only on the nature of the sorbate/polymer system and temperature. The second parameter *b* characterizes the deviation from the Henry-type behavior and is a measure of the isotherm curvature. The simplest Equation (8) for describing type III isotherms, called the anti-Langmuir equation [31], was proposed by Dubinin et al. [32]. Note that the equation in the form (8) was used by Kamiya et al. [33] to describe gas sorption in PDMS. The valuthe es of parameters are given in Table 1.

Since sorption of gases in polymers is exothermic (except for the quantum gases H_2_, He, Ne [34]), the amount of gas sorbed must increase with decreasing temperature. A deviation from this regularity was found for sorption in PTDMS. This deviation is most pronounced for methane, to a lesser extent for ethane, and even weaker for butane. Specifically, methane sorption in the polymer at 5 °C is less than at 10 °C. The same is observed in ethane at pressures below 6 atm (Figure 1). As follows from Table 1, the values of Henry’s constants for CH_4_ and C_2_H_6_ first increase with decreasing temperature from 35 to 10 °C and then decrease at 5 °C. In the case of *n*-butane, although its sorption increases with decreasing temperature from 35 to 5 °C, but Henry’s constants at 5 and 10 °C are quite close, that is, there is almost no difference in the sorption of *n*-butane at low pressures (<0.5 atm).

The only reason for the anomalous change in the solubility of the studied gases seems to be a change in the phase state of the polymer. Indeed, as noted in the Introduction, PTDMS silicon rubber contains long side chains that can crystallize when the temperature decreases, so that the polymer goes from an amorphous to a semi-crystalline state.

### 3.2. Thermal Behavior

**Figure 2:**
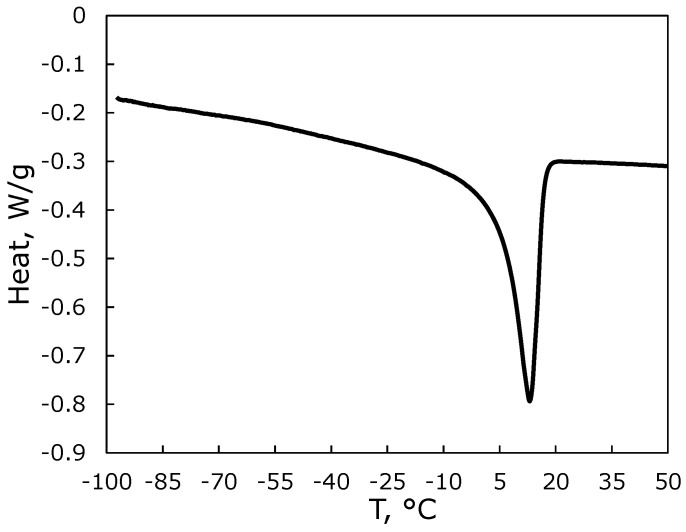
The DSC thermogram of poly(tetradecyl methyl siloxane).

Thermal analysis data indicate an endothermic transition associated with the melting of crystals composed of side alkyl chains in PTDMS. As shown in Figure 2, the single peak is observed for DSC thermogram of PTDMS in the temperature range studied. The pick melting temperature and latent heat of fusion of side-chains in the polymer are *T*_m_ = 12.9 °C and Δ*H*_f_ = 51.55 J/g.

The degree of crystallinity may be evaluated from the relation [35]:

(9) $$X_{c}=\Delta H_{f}/\Delta H_{f}^{0}$$

where $\Delta H_{f}^{0}$ is the heat of fusion for the 100% crystalline phase. With the value of $\Delta H_{f}^{0}=219.66$ J/g [19] or 222 J/g [23] the degree of crystallinity of PTDMS is $X_{c}\approx 0.23$. Molar mass of PTDMS unit is 256.5 g/mol. Conversion of Δ*H*_f_ into units of kJ/mol gives 13.2 kJ/mol. This value may be compared with the value of 14.1 kJ/mol for a similar polymer with the side-chain crystallinity, poly(tetradecyl acrylate), for which *T*_m_ = 19.5 °C [22]. O’Leary and Paul [22] have found that the minimum side-chain length needed for crystallization of the alkyl acrylate polymers is equal to 10. Note that for poly(*n*-alkyl methyl siloxane) rubbers, crystallization of the side chains was observed at the same minimum length of these chains [24].

### 3.3. Temperature Dependence of Solubility

The sorption enthalpy $\Delta H_{S}$ in the limit of low pressure may be evaluated with the following relation (the van’t Hoff equation):

(10) $$\frac{d\ln S_{0}}{d(1/T)}=-\frac{\Delta H_{S}}{R}$$

Given that sorption is exothermic ($\Delta H_{S}=\mathrm{const}<0$), ln *S*_0_ should increase strictly linearly with the reciprocal of absolute temperature. However, as previously shown (Figure 1a), the methane solubility in PTDMA is minimal at 5 °C, the lowest experimental temperature. In addition, the solubility of ethane at 5 °C is less than at 10 °C (at a pressure not exceeding 6 atm). These findings are inconsistent with the condition of exothermic sorption and are apparently related to the formation of a two-phase amorphocrystalline sorbent when the temperature is lowered to ~10 °C and below. The transition of amorphous rubber to a semi-crystalline state with decreasing temperature is confirmed by the DSC data presented above. Because of this, the application of Equation (10) should be limited to the temperature range in which the silicone rubber is in an amorphous state.

**Figure 3:**
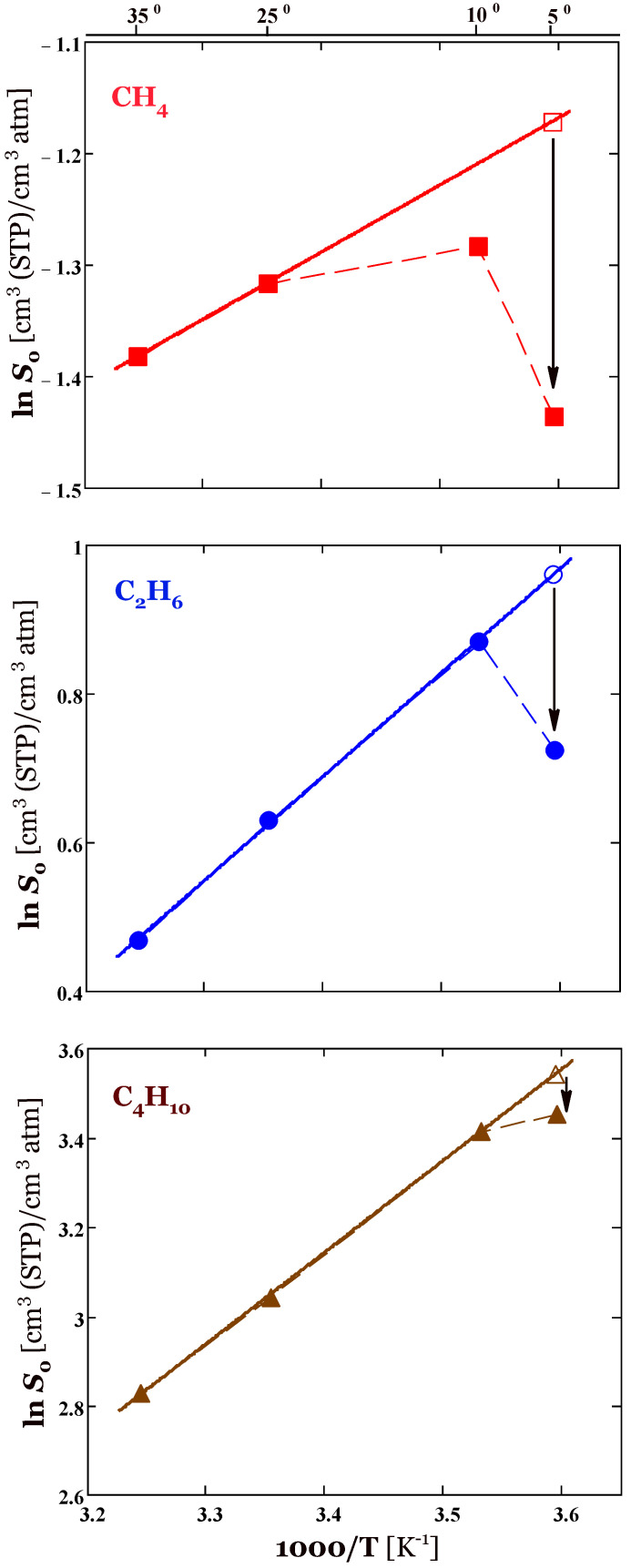
Temperature dependence of methane, ethane, and *n*-butane solubility coefficients in the limit *p* → 0. The filled symbols represent values of the Henry constants *S*_0_, solid lines represent the linear fits to the experimental data using the van’t Hoff relationship. The extrapolated solubility value at 5 °C is indicated by an open symbol for each sorbate. Arrows show the drop in gas solubility in a hypothetical amorphous rubber over that of semi-crystalline polymer at 5 °C.

A nearly perfect linear correlation of ln *S*_0_ with 1/*T* was found only for ethane and *n*-butane in the temperature range 10–35 °C (Figure 3). The sorption enthalpy is −11.6 and −17.1 kJ/mol for ethane and *n*-butane, respectively. At 5 °C, the solubility of ethane and *n*-butane deviates from linear fit, which probably indicates a change in the phase state of the polymeric sorbent. For methane, the situation is even more complicated, namely, the values of ln *S*_0_ at 35, 25, and 10 °C do not lie in one straight line. Therefore, in the case of methane, the enthalpy of sorption was determined from the solubility values at temperatures of 35 and 25 °C using the formula following from the van’t Hoff equation:

(11) $$\Delta H_{S}=R\frac{T_{1}T_{2}}{T_{1}-T_{2}}\ln\frac{S_{0}(T_{1})}{S_{0}(T_{2})}$$

where *T*_1_ and *T*_2_ are expressed in Kelvin. The calculated value of $\Delta H_{S}$ for the methane is equal −5.0 kJ/mol.

At 5 °C PTDMS is definitely in a semi-crystalline state. In this situation, according to Michaels and Bixler [36] the gas solubility in a two-phase semi-crystalline polymer may be expressed in simple form $S=S_{a}^{*}\varphi_{a}$ where $S_{a}^{*}$ is the solubility coefficient in a hypothetical, completely amorphous polymer. This simple model assumes that the gas is able to dissolve only in the amorphous phase of the polymer. The value of $S_{a}^{*}$ can be found by extrapolation of the amorphous-state solubility data to the temperatures of partially crystalline-state polymer. In our case, the value of $S_{a}^{*}$ was determined by extrapolating the dependence of ln *S*_0_ vs. 1/*T* to a temperature of 5 °C corresponding to a semi-crystalline polymer. The resulting hypothetical solubility values are shown by open symbols in Figure 3 and were 0.31, 2.61, and 34.53 cm^3^(STP)/(cm^3^·atm) for methane, ethane, and *n*-butane, respectively. Following the Michaels and Bixler model, it is possible to estimate the volume fraction of the amorphous phase in the polymer as

(12) $$\varphi_{a}={\left(S_{0}/S_{a}^{*}\right)}_{5^{0}C}$$

where *S*_0_ is the actual solubility of the penetrant in the polymer according to sorption data at 5 °C. We obtained *φ_a_* = 0.77, 0.79, and 0.92 for methane, ethane, and *n*-butane, respectively, and thus the volume fraction of the crystalline phase $\varphi_{c}=1-\varphi_{a}$ is 0.23, 0.21 and 0.08 for these penetrants.

The degree of crystallinity according to the thermal analysis of PTDMS was estimated above as *X_c_* ≈ 0.23. This means that the solubility of methane and ethane in semi-crystalline silicone rubber is adequately characterized by the model of Michaels and Bixler. In the case of *n*-butane, estimation by Equation (12) gives approximately 3 times lower polymer crystallinity compared to the result of thermal analysis. Apparently, this may mean that sorption sites in the crystalline domains or in the interphase layer between the crystalline and rubbery phases are available for *n*-butane, which has a high solubility in polysiloxanes compared to lighter alkanes.

### 3.4. Temperature Dependence of Diffusivity and Permeability

Diffusion coefficients of the gases in PTDMS were extracted from gravimetric data by the sorption half-time method. The permeability coefficients were further calculated as product of diffusivity and solubility coefficients, $P_{0}=D_{0}S_{0}$, where $D_{0}$ and $S_{0}$ are the diffusion and solubility coefficient in the limit of low pressure, respectively. The temperature dependence of diffusion and permeability is usually taken in the form of the Arrhenius equation:

(13) $$D_{0}(T)=D_{0}(\infty )e^{-E_{D}/RT},\;P_{0}(T)=P_{0}(\infty )e^{-E_{P}/RT}$$

where $E_{D}$ and $E_{P}$ is the diffusion and permeability activation energy, respectively. Because permeability is the product of solubility and diffusivity, the apparent activation energy of permeation $E_{P}=E_{D}+\Delta H_{S}$ where $\Delta H_{S}$ is the sorption enthalpy.

**Figure 4:**
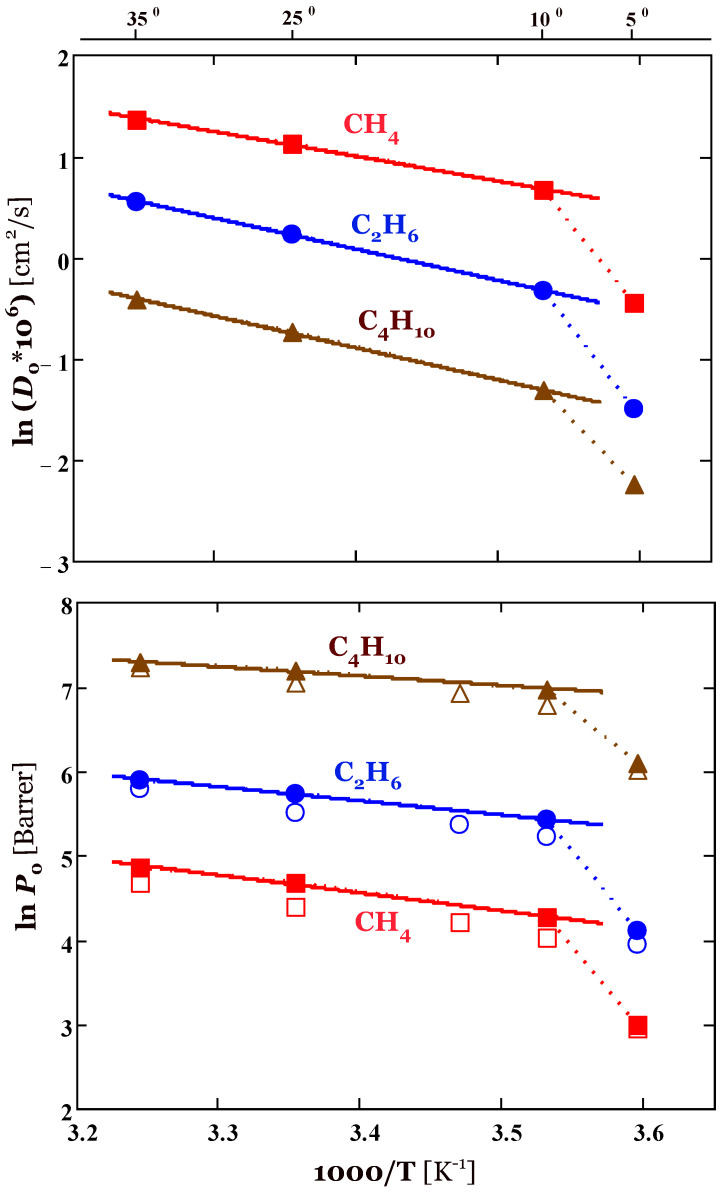
Temperature dependence of methane, ethane, and *n*-butane diffusivity *D*_0_ and permeability *P*_0_ in poly(tetradecyl methyl siloxane). The filled symbols represent values of *D*_0_ and *P*_0_ obtained by the sorption method, the solid lines represent the fits to diffusivity and permeability data using the Arrhenius-type relations (13) in the temperature range 10–35 °C. The open symbols show permeability values determined by the permeation method.

Diffusivity and permeability at various temperatures are presented in Table 2, and graphs are shown in Figure 4. As expected, the diffusivities increase in the order CH_4_ > C_2_H_6_ > *n*-C_4_H_10_ in accordance with the size of penetrant molecules. In the range 10–35 °C, the dependence of ln *D*_0_ versus 1/*T* is linear for all penetrants (Figure 4, top). The activation energies of diffusion are 18.6, 25.5, and 26.2 kJ/mol for methane, ethane, and *n*-butane, respectively. At 5 °C the values of diffusion coefficients deviate markedly downward from the linear dependence of ln *D*_0_ versus 1/*T*, indicating a significant decrease in the mobility of penetrant molecules in PTDMS due to crystallization of side alkyl chains. Namely, the diffusion coefficients of penetrants at 5 °C are 2.5–3 times lower compared to those at 10 °C (Table 2).

The permeability of alkanes decreases in the series *n*-C_4_H_10_ > C_2_H_6_ > CH_4_, that is, the change in permeability is controlled by the solubility of hydrocarbons in the silicone rubber (Figure 4, bottom). The dependence of ln *P*_0_ versus 1/*T* is linear in the temperature range 10–35 °C, the apparent activation energies of permeation are the positive and equal to 13.6, 13.9, and 9.1 kJ/mol for methane, ethane, and *n*-butane, respectively. This indicates that for all penetrants the permeability increases with increasing temperature. The behavior is significantly different from the temperature dependence of permeability in PDMS and POMS, in which only methane permeability increases with temperature, that is, *E_P_* > 0 only for methane. In these poly(siloxane)s, the permeability of condensable gases such as propane, *n*-butane, and even ethane drops with *T*, that is, *E_P_* < 0 [8,9,14]. The negative permeation activation energy is a consequence of the fact that the absolute value of the enthalpy of sorption is greater than the diffusion activation energy for these alkanes. By contrast, the activation energy of diffusion dominates the transport in PTDMS ($E_{D}>-\Delta H_{S}$), which leads to a positive *E_P_* value for all alkanes studied. Thus, substitution of methyl groups in PDMS by long *n*-alkyls leads to changes in the transport properties of silicon rubbers: increasing the diffusion barrier (increase *E_D_*) and weakening of sorbate/polymer interaction energy (reduction of heat effect) and, as a consequence, to significant positive values of activation energy of permeation. As with diffusivities, there is a marked decrease in penetrant permeabilities at 5 °C, which seems to be associated with the side-chain crystalline structure of the silicone rubber. Specifically, the permeabilities of penetrants at 5 °C are 2.4–3.7 times lower compared to those at 10 °C (Table 2).

**Figure 5:**
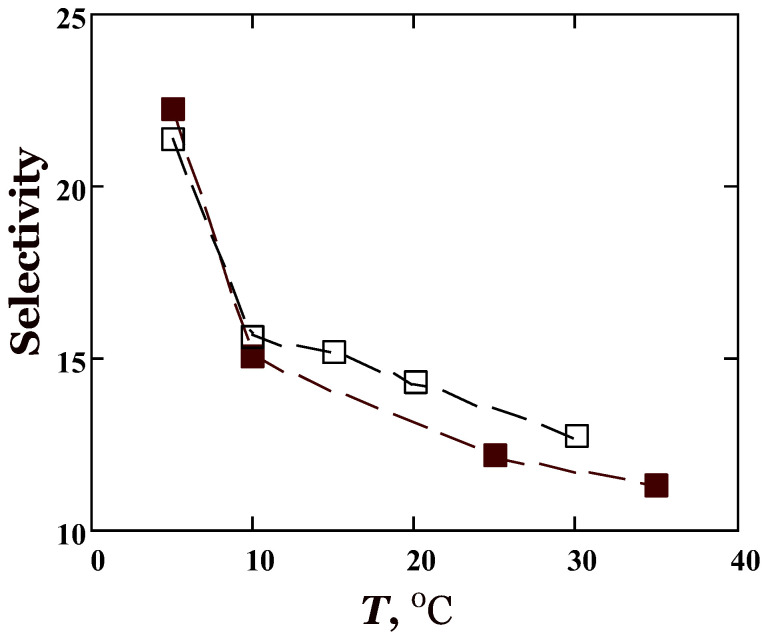
Temperature dependence of *n*-butane/methane selectivity of poly(tetradecyl methyl siloxane) in the zero pressure limit. The filled symbols represent values obtained by the sorption method, the open symbols show selectivity values determined by the permeation method.

Figure 4 (bottom) shows permeability results obtained by two independent methods, the sorption method (filled symbols) and the constant volume/variable pressure method (open symbols). The permeability values obtained by the sorption method are somewhat higher (by 10–20%) compared to the steady-state permeability measurements. Possible reasons for the discrepancy in the results of the two methods for determining the transport properties of membranes were discussed in particular by Friess et al. [37]. In general, taking into account measurement errors, the acceptable agreement between the permeability coefficients from sorption and permeation methods were obtained. Note that the agreement between these methods gets better for the ratio of permeabilities of *n*-butane to methane, that is, for *n*-butane/methane selectivity (Figure 5). Interestingly, selectivity increases with temperature not only for the amorphous state of the membrane, but also at lower temperatures (below 10 °C) when crystallization of the side chains in PTDMS occurs.

The *n*-butane/methane selectivity *α* and its components, the sorption selectivity $\alpha_{S}$ and the diffusion selectivity $\alpha_{D}$, were calculated as

(14) $$\alpha_{S}=S_{0}(C_{4}H_{10})/S_{0}({\mathrm{CH}}_{4}),\;\alpha_{D}=D_{0}(C_{4}H_{10})/D_{0}({\mathrm{CH}}_{4}),\;\alpha =P_{0}(C_{4}H_{10})/P_{0}({\mathrm{CH}}_{4})$$

One can see from Table 2 that PTDMS silicone rubber is a sorption-selective polymer, that is, $\alpha_{S}>\alpha >\alpha_{D}$. Note also that the *n*-C_4_H_10_/CH_4_ selectivity increases along with sorption selectivity with decreasing temperature. A similar increase in the *n*-C_4_H_10_/CH_4_ selectivity with decreasing temperature is observed for PDMS membranes [8,9] and is associated with the opposite temperature dependence of the permeability for methane and *n*-butane. The permeability of methane in PDMS increases with temperature, whereas the permeability of *n*-butane decreases with temperature (*E_P_* > 0 for CH_4_, but *E_P_* < 0 for *n*-C_4_H_10_). A quite different situation was observed for the polymer under consideration. As demonstrated above, the permeability of CH_4_ and *n*-C_4_H_10_ in PTDMS increases with temperature, that is, the apparent permeability activation energies are positive for the studied hydrocarbons. However, the temperature dependence of the permeability of *n*-butane is less pronounced (*E_P_* = 9.1 kJ/mol) compared to methane (*E_P_* = 13.6 kJ/mol), which provides an increase in the *n*-C_4_H_10_/CH_4_ selectivity with decreasing temperature. An additional feature of gas transport in PTDMS is the change in its phase state at temperatures near and below 10 °C, when PTDMS undergoes partial crystallization. Crystallization of the side chains is accompanied by a decrease in the permeability of both methane and butane, but this decrease is less for butane. The probable explanation is related to the availability to condensable *n*-butane (*T_b_* ≅ 0 °C), not only the amorphous but also, to a certain extent, the crystalline phase of the polymer.

## 4. Conclusions

Silicone rubber, namely, crosslinked poly(tetradecyl methyl siloxane) (PTDMS) was synthesized. The transport properties of PTDMS with respect to CH_4_, C_2_H_6_, and *n*-C_4_H_10_ alkanes were studied in the temperature range 5–35 °C.

Linear sorption isotherms were obtained for methane. The isotherms for ethane and *n*-butane are convex toward the pressure axis, and the curvature of the isotherms increases as the temperature decreases. Contrary to convenient rubber or glassy polymers, for which gas solubility increases with decreasing temperature, an abnormal temperature dependence of gas sorption is observed for synthesized polymer. Specifically, the sorption of CH_4_ at 5 °C is less than the sorption at higher temperatures over the entire pressure range (up to 10 atm), and the sorption of C_2_H_6_ at 5 °C is less than the sorption at 10 °C at pressures below 6 atm. The abnormal behavior may be attributed to side-chains crystallization of the polymer at temperatures below ~10 °C, as confirmed by thermal analysis data.

The degree of crystallinity from thermal analysis (23%) is consistent with the volume fraction of crystallinity which was evaluated by the two-phase model of Michaels and Bixler for methane and ethane in PTDMS. For *n*-butane this model shows a small value of crystallinity (8%), which is probably due to the possibility of sorption of *n*-butane molecules not only in the amorphous but also in the crystalline phase of the polymer.

Permeability measured by independent techniques, namely, the sorption and permeation methods, decrease in the order *n*-C_4_H_10_ > C_2_H_6_ > CH_4_. The solubility of these alkanes in PTDMS changes in the same order. This indicates that PTDMS is the sorption-selective membrane. In contrast to poly(dimethylsiloxane), PTDMS displays an increase in permeability with temperature for the alkanes studied, including *n*-butane. This means that, for this silicon rubber, the activation energy of diffusion is greater than the heat of sorption. In the semi-crystalline state of the polymer, gas diffusion and permeability are markedly reduced compared to the amorphous state of the polymer. Nevertheless, the *n*-C_4_H_10_/CH_4_ selectivity reaches its greatest value in the semi-crystalline state of PTDMS and is 23 at 5 °C. Future work is needed for determining the permeation properties of poly(siloxane)s with crystallizable side chains in mixed-gas conditions.

## Figures and Tables

**Table 1:** Fitting parameters of Equation (8) for CH_4_, C_2_H_6_ and *n*-C_4_H_10_ at different temperatures ([*S*_0_)] = cm^3^(STP)/(cm^3^·atm), [*b*] = atm^−1^). The values of the correlation coefficient (*R*^2^) are also given.

*T*, °C	Methane *		Ethane			*n*-Butane		
	*S*_0_	*R* ^2^	*S*_0_	*b*	*R* ^2^	*S*_0_	*b*	*R* ^2^
5	0.238	0.9998	2.062	0.039	0.9959	31.601	0.721	0.9984
10	0.277	0.9999	2.387	0.018	1.0	30.368	0.606	0.9980
25	0.268	1.0	1.874	0.011	1.0	20.946	0.334	0.9994
35	0.251	0.9997	1.597	0.010	1.0	16.892	0.223	0.9994

* For methane the *b* parameter in Equation (8) was considered to be zero.

**Table 2:** Hydrocarbon diffusivity and permeability in PTDMS in the limit pressure *p* → 0. Effect of temperature on *n*-C_4_H_10_/CH_4_ solubility (*α*_S_), diffusivity (*α_D_*) and permeability (*α = α*_S_·*α*_D_ ) selectivities are also shown.

*T*, °C	*D*_0_·10^6^ (cm^2^/s)			*P*_0_ (Barrer)			*α*_S_	*α_D_*	*α*
	CH_4_	C_2_H_6_	*n*-C_4_H_10_	CH_4_	C_2_H_6_	*n*-C_4_H_10_			
5	0.64	0.22	0.11	20	61	445	132.8	0.17	22.6
10	1.95	0.72	0.27	71	226	1070	109.6	0.14	15.3
25	3.06	1.25	0.48	108	308	1315	78.2	0.16	12.5
35	3.91	1.73	0.66	129	364	1462	67.3	0.17	11.4

## Data Availability

Not applicable.
